# Temporal Data Set Reduction Based on D-Optimality for Quantitative FLIM-FRET Imaging

**DOI:** 10.1371/journal.pone.0144421

**Published:** 2015-12-11

**Authors:** Travis Omer, Xavier Intes, Juergen Hahn

**Affiliations:** 1 Department of Biomedical Engineering, Rensselaer Polytechnic Institute, Troy, NY, United States of America; 2 Department of Chemical & Biological Engineering, Rensselaer Polytechnic Institute, Troy, NY, United States of America; Irvine, UNITED STATES

## Abstract

Fluorescence lifetime imaging (FLIM) when paired with Förster resonance energy transfer (FLIM-FRET) enables the monitoring of nanoscale interactions in living biological samples. FLIM-FRET model-based estimation methods allow the quantitative retrieval of parameters such as the quenched (interacting) and unquenched (non-interacting) fractional populations of the donor fluorophore and/or the distance of the interactions. The quantitative accuracy of such model-based approaches is dependent on multiple factors such as signal-to-noise ratio and number of temporal points acquired when sampling the fluorescence decays. For high-throughput or *in vivo* applications of FLIM-FRET, it is desirable to acquire a limited number of temporal points for fast acquisition times. Yet, it is critical to acquire temporal data sets with sufficient information content to allow for accurate FLIM-FRET parameter estimation. Herein, an optimal experimental design approach based upon sensitivity analysis is presented in order to identify the time points that provide the best quantitative estimates of the parameters for a determined number of temporal sampling points. More specifically, the D-optimality criterion is employed to identify, within a sparse temporal data set, the set of time points leading to optimal estimations of the quenched fractional population of the donor fluorophore. Overall, a reduced set of 10 time points (compared to a typical complete set of 90 time points) was identified to have minimal impact on parameter estimation accuracy (≈5%), with *in silico* and *in vivo* experiment validations. This reduction of the number of needed time points by almost an order of magnitude allows the use of FLIM-FRET for certain high-throughput applications which would be infeasible if the entire number of time sampling points were used.

## Introduction

Fluorescence techniques have been applied to a broad range of biomedical research problems for over 100 years [1]. One of the benefits of their nondestructive, highly-sensitive and noninvasive nature is that they can be used on living samples [2], reducing the complexity and cost of many experiments involving biological systems. Fluorescence imaging can be implemented based on various contrast types, though fluorescence lifetime imaging (FLIM) has proven especially beneficial in biological systems [3–5]. Fluorescence is typically induced using high-speed lights or lasers which cause electrons in the fluorescent molecule to attain higher energy states. They eventually return to their ground state and in the process can emit a specific wavelength and profile of light. The average time that the molecule remains in the excited state is deemed the fluorescence lifetime, is usually short-lived—up to nanoseconds in duration [6]—and independent of the measurement method. The difference in fluorescence lifetime between molecules and local environments of a sample provides contrast to the image.

One particularly useful implementation of FLIM is Förster resonance energy transfer (FLIM-FRET). In the case of FLIM-FRET, estimation of fluorescence lifetime and FRET donor populations can be used to provide insight into cellular signaling events [7, 8], cell-cell adhesion [9, 10] or apoptosis [11] to name a few. Techniques for measuring these phenomena are generally separated into two groups: time domain and frequency domain. In each case, modulated lights or lasers are used to excite the sample. In frequency domain methods, the amplitude and phase of the resulting fluorescence is measured and used to estimate the parameters of interest. Alternatively, time domain methods record the resulting fluorescence at different time delays relative to the excitation pulse and build up histograms used to determine decay parameters. Frequency domain methods tend to have better results at high intensities [2] while time domain methods tend to have better signal-to-noise ratios [12]. Herein, we focus on time domain methods as improved signal-to-noise ratios are especially useful for *in vivo* and high-throughput applications which are photon starved.

In time domain FLIM-FRET, parameters are typically estimated by fitting a biexponential model to recorded FLIM-FRET data, which can be a challenging procedure if good estimates need to be obtained. In order to address this problem, dense temporal sampling is commonly acquired. These comprehensive temporal data sets result in accurate parameter estimates, but at the cost of increased imaging time, limiting the applicability to relatively few *in vivo* or high-throughput applications. Recently, methods such as rapid lifetime determination (RLD) [13, 14] and phasor analysis [15, 16] have gained popularity as they circumvent the need for iterative fitting based on large temporal data sets. These non-fitting methods directly calculate parameters of interest such as fluorescence lifetime and FRET fractions. In RLD, the recorded decay curve is sectioned into separate regions. The areas under these regions are then used to calculate the parameters of interest. A major benefit of RLD is the real-time speed at which these calculations can be carried out. However, RLD is accurate only when the instrument response function (IRF) of the system is negligible compared to the lifetime imaged. In the case of short lifetime such as encountered when using near-infrared fluorophores, the accuracy in estimations is compromised. This effectively restricts the application of RLD to *in vitro* applications and visible fluorophores.

In phasor analysis, the recorded decay curve is transformed into a vector-like representation within a unit semicircle using sine-cosine transforms [17]. Each pixel in a recorded image is converted into a phasor whose location in the semicircle is determined by its decay information. Because each fluorescent molecule has a phasor associated with it, the relative abundances of each fluorophore can be identified from the phasor plot. Phasor analysis, however, is less accurate than fitting at low photon counts [18] which also limits its applicability for *in vivo* techniques. Therefore, there is still a need for improved acquisition and analysis methods for *in vivo* and high-throughput FLIM-FRET applications.

Previous work [19] sought to solve this problem by optimizing the data acquired by means of *in silico* large, random trials. It was determined that it is possible to significantly reduce the acquisition time while still taking advantage of the low-light benefits of time domain FLIM-FRET. However, that work relies upon a computationally expensive method which is chiefly applicable to well-plate, microscopy imaging applications. Small changes to experimental conditions require repeating extensive random trials that become intractable with increasingly complex experimental systems. Additionally, cutting-edge implementations of FLIM-FRET platforms include multispectral/hyperspectral [20–22] information and require a more elegant solution due to the increased dimensionality. Experimental design, which is widely used in process engineering [23], pharmacology [24] and other imaging modalities, especially magnetic resonance imaging [25], provides a solution to this problem. These methods determine the optimal experimental conditions needed to obtain the maximum information content from the data. Herein, the D-optimality criterion, which is a commonly used metric in optimal experimental design [26], is applied to the Fisher Information matrix and used to obtain a smaller, information-rich set of data that results in decreased imaging time without a significant loss in estimation accuracy. This approach presents the first of its kind to reduce FLIM-FRET data acquisition requirements using an experimental design framework. The results are compared to previous work and validated via *in vivo* experiments. This decrease in imaging acquisition time facilitates the analysis of *in vivo* and high-throughput FLIM-FRET applications.

## Methods

### FLIM-FRET

One of the main instrumental techniques of measuring FLIM-FRET is via a time-gated imaging acquisition. Briefly, a femtosecond laser is used to excite the sample. The fluorescence emission is then collected by a camera which is synchronized with the laser excitation. To capture temporal information, the camera is equipped with a gating system (shutter) that is open at a precise time delay after the laser burst and only for small period of time (gate width). This acquisition sequence is repeated for different time delays relative to the laser in order to acquire a number of sequential time gates that sample the fluorescence decay curve. From these temporal, lapsed fluorescence images, parameters of interest such as the fluorescence lifetime and the fraction of fluorophores undergoing FRET can be extracted.

FLIM-FRET leverages the phenomenon of nonradiative energy transfer between two fluorophores to locate and quantify cell signaling processes, protein-protein interactions and other nanometer range events [27–29]. These two carefully selected fluorophores are characterized as a “donor” and an “acceptor”. The donor has an emission wavelength within the excitation spectrum of the acceptor. When the acceptor fluorophore is within approximately 10nm [30] of the donor fluorophore, an excited donor can transfer some of its energy to the acceptor resulting in a measurable reduction (quenching) in fluorescence lifetime of the donor [31]. The resulting fluorescence decay from this phenomenon is most often modeled using a biexponential equation

$$\begin{array}{c} I=A_{1}e^{\frac{-t}{\tau_{1}}}+A_{2}e^{\frac{-t}{\tau_{2}}}, \end{array}$$ (1)

where the parameters are A_1_, the quenched donor fraction, A_2_, the unquenched donor fraction, *τ*_1_, the lifetime of the quenched donor fraction and *τ*_2_ the unquenched lifetime of the donor. In the case of FRET, A_1_ + A_2_ = 1 which reduces the number of parameters to three. Due to the time resolution of the measurement system, however, an additional term, the instrument response function (IRF), is needed in order to fit the data more accurately. Therefore, what is actually recorded by the system is more accurately modeled by the biexponential equation convolved with the IRF of the system

$$\begin{array}{c} I=IRF\left(t\right)⊗(A_{1}e^{\frac{-t}{\tau_{1}}}+A_{2}e^{\frac{-t}{\tau_{2}}}), \end{array}$$ (2)

where (⊗) represents the convolution operator.

**Fig 1:**
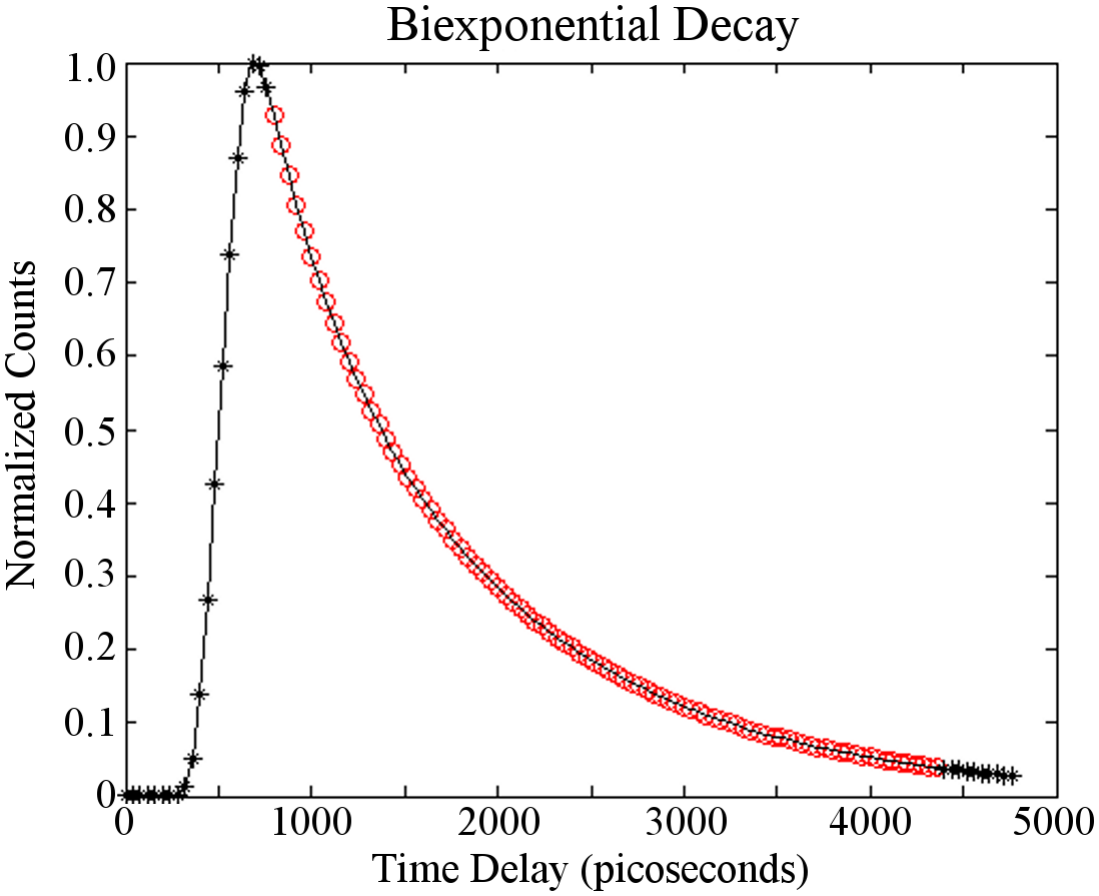
A synthetic TPSF from a biexponential model. A synthetic TPSF showing the possible position of 120 collected time points. Note that approximately 90 time points (red circles) fall within a useful range for estimating fluorescence lifetime parameters for this case.

In order to guarantee accurate estimates of these parameters many time gates are often collected [32] and constructed into a temporal point spread function (TPSF) as shown in Fig 1. Early time gates in the rising portion of the TPSF provide means to impart spatial resolution [33, 34] whereas later time gates in the decay portion of the TPSF are most helpful in estimating fluorescence lifetime parameters [35]. The remainder of this work uses these 90 equally-spaced (every 40 psec) time gates corresponding to the decay portion of the TPSF.

### Sensitivity Analysis

Local sensitivity can be defined as the partial derivative of the output of a system with respect to a parameter [36]. Herein the direct differentiation method is used to find the sensitivity which can be defined as

$$\begin{array}{c} s\left(t\right)=\frac{\partial y(t)}{\partial \theta_{i}}, \end{array}$$ (3)

where y is the output and *θ*_*i*_ is the *i* ^th^ parameter (from a total of *p* parameters). The sensitivity is a function of time (*n* time points) and can be written in vector format. One sensitivity vector is constructed for each parameter

$$\begin{array}{c} s_{i}={\left[\frac{\partial y(t)}{\partial \theta_{i}},\ldots ,\frac{\partial y(t)}{\partial \theta_{i}}\right]}^{T}, \end{array}$$ (4)

which is of size *n* × 1. The sensitivity matrix can then be constructed from each of the *p* sensitivity vectors creating a sensitivity matrix of size *n* × *p*,

$$\begin{array}{c} S=[s_{1},\ldots ,s_{p}]. \end{array}$$ (5)

The Fisher information matrix (FIM)—the inverse of which provides the Cramér—Rao lower bound—provides a comparison of the quality of experimental designs [37]. Assuming uncorrelated measurement noise that is constant with time, the FIM can be defined as the product of the transpose of the sensitivity matrix with itself

$$\begin{array}{c} FIM=S^{T}S, \end{array}$$ (6)

which is a *p* × *p* matrix, i.e., for three parameters, the FIM becomes a 3 × 3 matrix. The FIM is used extensively in experimental design procedures and is frequently used in the study of biological systems [38].

### *In Silico* Experiment

Synthetic decay curves were generated using the biexponential model shown in Eq (2) and a variety of parameter values (*A*_1_ = 0.1 − 0.9, *τ*_1_ = 250 − 450 *psec*, *τ*_2_ = 1200 *psec*). These lifetime values correspond to the quenched and unquenched lifetimes of the near-infrared (NIR) FRET pair Alexa Fluor 700 (AF700)—Alexa Fluor 750 (AF750). This pair is well suited for high-throughput *in vitro* and *in vivo* applications due to the minimal auto-fluorescence and attenuation at these wavelengths [39, 40] and significant lifetime reduction of the donor upon FRET (AF700) [41]. 100 decay curves were generated at each set of parameter values. Poisson noise is most frequently used to represent the noise present in the imaging process [32, 42] and was added to each of the curves.

### *In Vivo* Experiment

A full description of the imaging protocol can be found in Zhao et al [43]. Briefly, an athymic nude female mouse was injected with transferrin labeled AF700 and AF750 in RPMI 1,640 media at molar ratios of 2:1 via the tail vein. 24 hours post-injection the mouse was imaged using a wide-field illumination method with a spectral filter to restrict recorded light to the wavelengths of AF700 emission. The image was cropped to include only the areas of interest (172 × 128 pixels), which include the bladder and tumor of the mouse. As 120 time gates were collected, the complete dataset is a 172 × 128 × 120 matrix. A TPSF (see Fig 1) was constructed for each pixel of this data and parameter estimation was performed across the image. A bright field image was also acquired and overlaid to provide context of the results.

All animal protocols were conducted with approval by the Institutional Animal Care and Use Committee, Rensselaer Polytechnic Institute. Tumor sizes were monitored throughout the experiment and the maximum allowed tumor volume was 0.5 cm^3^. The animals were monitored for significant weight loss (over 15% of initial body weight) or significant aversion to feeding, grooming, drinking or eating. These and other indicators, such as signs of pain, illness, tumor ulceration or distress were evaluated by an attending veterinarian to determine if they should undergo euthanasia. The animals were imaged while under vapor anesthesia using isoflurane and monitored using a physiological monitoring system (oxygen saturation, heart rate and breathing rate). Body temperature was maintained by an air warmer during the imaging session and monitored using a rectal thermometer. Depth of anesthesia was checked at 3-5 minute intervals by observing respiratory rate and response to toe pinch. Animals were euthanized by carbon dioxide inhalation for at least 60 seconds. Clinical death was determined by no sign of respiration and no movement. The death was confirmed by cervical dislocation or decapitation.

### Experimental Design Criteria

Experimental design methods seek to determine the best set of conditions for maximizing information content of the data in some form. In order to directly compare different experimental designs, a scalar measure of a design matrix is needed. There are many available measures such as the A-optimality criterion, D-optimality criterion or E-optimality criterion among others [44, 45]. Though each has its benefits, the D-optimality criterion is especially useful in parameter estimation problems and will be used herein. It is defined as

$$\begin{array}{c} \phi D=max\;det(FIM). \end{array}$$ (7)

The D-optimality criterion seeks to maximize the determinant of the information matrix which is equivalent to minimizing the volume of the confidence region for the parameter estimates. D-optimal designs are robust and generally produce satisfactory results even given poor initial parameter estimates as well as being independent of the scale of the variables of the model [46].

### Application to FLIM-FRET

It is the goal of this work to formulate an optimal experimental design procedure that can be used to determine the number and location of time gates used for FLIM-FRET imaging. The model shown in Eq (2) is used and it is the goal to estimate its parameters accurately using as little data that needs to be measured as possible. It should be noted that for the purpose of this paper, rich data sets were collected to allow for a comparison of the estimation accuracy of a reduced data set to the full set. However, once the procedure developed in this paper is established, it is possible to collect a rich data set only for a small imaging region, perform the experimental design, and then acquire data for a larger region to be imaged using the reduced number of time gates. The partial differentials of *I* with respect to each parameter can be written as

$$\begin{array}{ccc} \frac{\partial I(t)}{\partial A_{1}} & = & IRF\left(t\right)⊗(e^{\frac{-t}{\tau_{1}}}-e^{\frac{-t}{\tau_{2}}})\\ \frac{\partial I(t)}{\partial \tau_{1}} & = & IRF\left(t\right)⊗(\frac{A_{1}te^{\frac{-t}{\tau_{1}}}}{\tau_{1}^{2}})\\ \frac{\partial I(t)}{\partial \tau_{2}} & = & IRF\left(t\right)⊗(\frac{\left(1-A_{1}\right)te^{\frac{-t}{\tau_{2}}}}{\tau_{2}^{2}}). \end{array}$$ (8)

By combining Eqs (4), (5) and (8), the sensitivity matrix of the system can be constructed

$$\begin{array}{c} S=[\begin{array}{ccc} \frac{\partial I(t_{1})}{\partial A_{1}} & \frac{\partial I(t_{1})}{\partial \tau_{1}} & \frac{\partial I(t_{1})}{\partial \tau_{2}}\\ & \vdots & \\ \frac{\partial I(t_{n})}{\partial A_{1}} & \frac{\partial I(t_{n})}{\partial \tau_{1}} & \frac{\partial I(t_{n})}{\partial \tau_{2}} \end{array}]. \end{array}$$ (9)

For *n* available time gates this becomes an *n* × 3 matrix which can be used to define the FIM according to Eq (6). A helpful property of this formulation of the FIM is that it satisfies the superposition principle. For example, the sum of the FIM from time gates 1 and 2 (FIM[1] + FIM[2]) is equal to the FIM calculated using both time gates simultaneously (FIM[1, 2]), or more generally:

$$\begin{array}{c} FIM[a]+\ldots +FIM[z]=FIM[a,\ldots ,z]. \end{array}$$ (10)

This property proves useful as it allows the optimization problem to be written as a linear combination of FIMs as shown in the next section.

### Optimization Problem

The optimization problem is constructed by using Eq (9) and leveraging the superposition property shown in Eq (10). A binary vector *χ* (i.e., *χ*_*i*_ ∈ 0, 1, *i* = 1, 2, …, *n*) which represents the set of time gates chosen for analysis, makes it possible to pre-calculate the FIM for each individual time gate. The optimization problem then determines the optimal combination of time gates by combining the different sets of FIMs. It should be noted that it is only necessary to pre-compute one FIM for each time gate that can be measured and that the FIMs resulting from a set of time gate measurements directly follow from

$$\begin{array}{c} FIM=\sum_{i=1}^{n}\chi_{i}\;FIM\left[i\right]=\sum_{i=1}^{n}\chi_{i}S_{i}^{T}S_{i}. \end{array}$$ (11)

The complete optimization problem then becomes

$$\begin{array}{ccc} & & \underset{x}{\text{maximize}}\;det(\sum_{i=1}^{n}\chi_{i}S_{i}^{T}S_{i})\\ & & \text{subject}\;\text{to}\;\chi_{i}\in \left\{0,1\right\}\;i=1,2,\ldots ,n.\\ & & \;\;\;\sum_{i=1}^{n}\chi_{i}\leq r, \end{array}$$ (12)

where *r* is the maximum number of time gates to be selected and *n* is the number of total available time gates. The goal is to retain a combination of time gates that results in as good estimation accuracy as possible for a reduced number of time gates. Fewer time gates results in a less demanding data acquisition, which reduces the acquisition time. The exact number of time gates needs to be determined by repeatedly solving this optimization problem for different values of r and determining further reduction of *r* will result in significant changes of the optimality criterion. The simplest, yet most inefficient, way of solving this optimization problem is an exhaustive search of all possible solutions. This is a combinatorial problem with total combinations (*C*) described by the binomial coefficient

$$\begin{array}{c} C=\frac{n!}{(n-r)!r!}, \end{array}$$ (13)

where *r* time gates are chosen from *n* possibilities. This brute force approach is manageable for a small number of time gates, e.g., choosing up to 5 time gates from the total of 90 (4 × 10^7^ combinations); however, enumeration for a larger number of time gates becomes intractable. Suboptimal methods for approximating the solution of this optimization problem exist. For example, it is possible to solve the optimization problem by selecting the first time gate which maximizes the optimization problem. Next, time gates are sequentially added by calculating which remaining time gates best complement the current set resulting in a larger function value. The scale of the sequential combinations (*SC*) is orders of magnitude smaller and is described by the product

$$\begin{array}{c} SC=\sum_{i=0}^{r-1}\left(n-i\right), \end{array}$$ (14)

for a subset of *r* time gates chosen from *n* total time gates. This results in only 440 combinations, for example, for choosing 5 time gates from a total of 90. While such an approach can significantly reduce the computational burden of solving this problem, there is no guarantee, and in fact it is unlikely, that sequentially selecting the best *r* time gates will result in the same set as when they are chosen optimally. The best solution is to solve this mixed integer non-linear problem (MINLP) using a MINLP solver. The solution will be significantly faster than an exhaustive search of the solution space while ensuring that the results will be optimal. Further, it can be shown that the optimization problem resulting from maximizing the determinant of a positive semi-definite matrix (like the FIM) is convex [47, 48] which ensures a global solution to the problem. Herein, the branch and bound algorithm which is included in the Basic Open-source Nonlinear Mixed Integer (BONMIN) [49] solver was used to solve the optimization problem. The branch and bound algorithm treats the solution set as a rooted tree. Branches of this tree can then be pruned by relaxing the integer constraints of the problem and calculating upper and lower bounds on optimal values for that branch. Any branch with less desirable bounds is pruned and the solution set is reduced is size. In the worst case scenario the branch and bound algorithm iterates through the entire solution set. However, in practice, much of the solution set is pruned and it converges to a solution in seconds or minutes as opposed to hours or days.

A nominal set of experimental values (*A*_1_ = 0.3, *τ*_1_ = 300 *psec*, *τ*_2_ = 1200 *psec*) similar to those used in the *in silico* experiment were selected to calculate a set of FIMs. Table 1 shows the optimal function values and set of optimum time gates after solving the optimization problem shown in Eq (12) using this set. The minimum number of time gates examined is three as this is the number of parameters to be estimated. For brevity, the solutions are shown for only up to ten time gates, however, solutions for larger sets of time gates are easily obtained.

**Table 1:** Optimization results for nominal values. Optimization results using sensitivity analysis via BONMIN and three fitted parameters. The location of the time gates are reported as the delay (in picoseconds) after excitation.

# of Time Gates	Opt. Func. Value	Set of Time Gates
3	0.0097	160, 1320, 3600
4	0.0194	160, 1320, 1360, 3600
5	0.0387	160, 200, 1320, 1360, 3600
6	0.0757	160, 200, 1320, 1360, 3560, 3600
7	0.1135	160, 200, 1320, 1360, 1400, 3560, 3600
8	0.1700	160, 200, 240, 1320, 1360, 1400, 3560, 3600
9	0.2496	160, 200, 240, 1320, 1360, 1400, 3520, 3560, 3600
10	0.3325	160, 200, 240, 1320, 1360, 1400, 1440, 3520, 3560, 3600

The optimal function value and optimal time points were verified via an exhaustive search of all combinations for sets of three, four and five time gates. Further verification of larger sets was infeasible due to the computational effort required. There is a clear trend in which optimal time points are clustered into three groups—one near the beginning, one in the middle and one at the end of the available set of time gates. In each of the cases shown in Table 1, the optimization problem was solved in fewer than 3 minutes using a desktop PC (3.4 GHz Core-i7, 16GB RAM). Obtaining results using the previous method of large, random trials typically required millions of iterations and between 24-48 hours to complete using the same hardware. Using the new framework, various experimental conditions can be examined in a fraction of the time. For example, Table 2 contains a few samples of other useful FRET pairs along with their nominal and quenched lifetimes and quenched donor fraction. The optimal set of time gates is obtained in less than 10 seconds for each of the FRET pairs. In each case, the general trend of groups of early, intermediate and late time gates continues. As expected, however, the exact position of the time gates varies depending on the experimental conditions.

**Table 2:** Optimization results of additional FRET pairs. A comparison of three different FRET pairs, their nominal and quenched (short) lifetimes, an example quenched donor fraction and the calculated optimal time gates using the framework developed herein.

FRET Combo	Nominal Lifetime	Short Lifetime	Donor Fraction	Optimal Time Gates
CFP-YFP [50, 51]	2.5 ns	1.6 ns	0.3	480, 560, 600, 1800, 1840, 2160, 2400, 3320, 3400, 3520
EGFP-mRFP1 [52]	2.2 ns	0.95 ns	0.1	440, 480, 520, 560, 600, 2040, 2080, 2440, 3560, 3600
TagGFP-TagRFP [53]	2.2 ns	0.95 ns	0.1	480, 560, 600, 1800, 1840, 2160, 2400, 3320, 3400, 3520

## Results and Discussion

One of the exciting applications of FLIM-FRET is in drug discovery [54, 55]. For example, FLIM-FRET enables the visualization and quantification of targeted drug delivery of therapeutic agents to neoplastic tissues [56–58]. For instance, Abe et al. [59] demonstrated the potential of FLIM-FRET in measuring target engagement *in vivo* in breast tumors using NIR FRET pairs labeled with transferrin. Because the transferrin receptor is homodimeric (i.e. two transferrin molecules bind to the receptor within 2-10nm of each other), FRET pairs can be conjugated to the transferrin and used to determine when it is bound at the plasma membrane and undergoing endocytosis (represented by a FRET positive signal) [60–62]. In drug discovery applications, this FRET positive signal is of particular interest as it allows the quantification of internalized [43, 59, 63] transferrin-labeled molecules. When this process is represented by the biexponential model shown in Eq (2), the parameter of most interest is the quenched donor fraction, A_1_. *In silico* and *in vivo* experiments based upon this drug delivery method are used to validate the optimization results and estimate A_1_.

### *In Silico* Results

**Fig 2:**
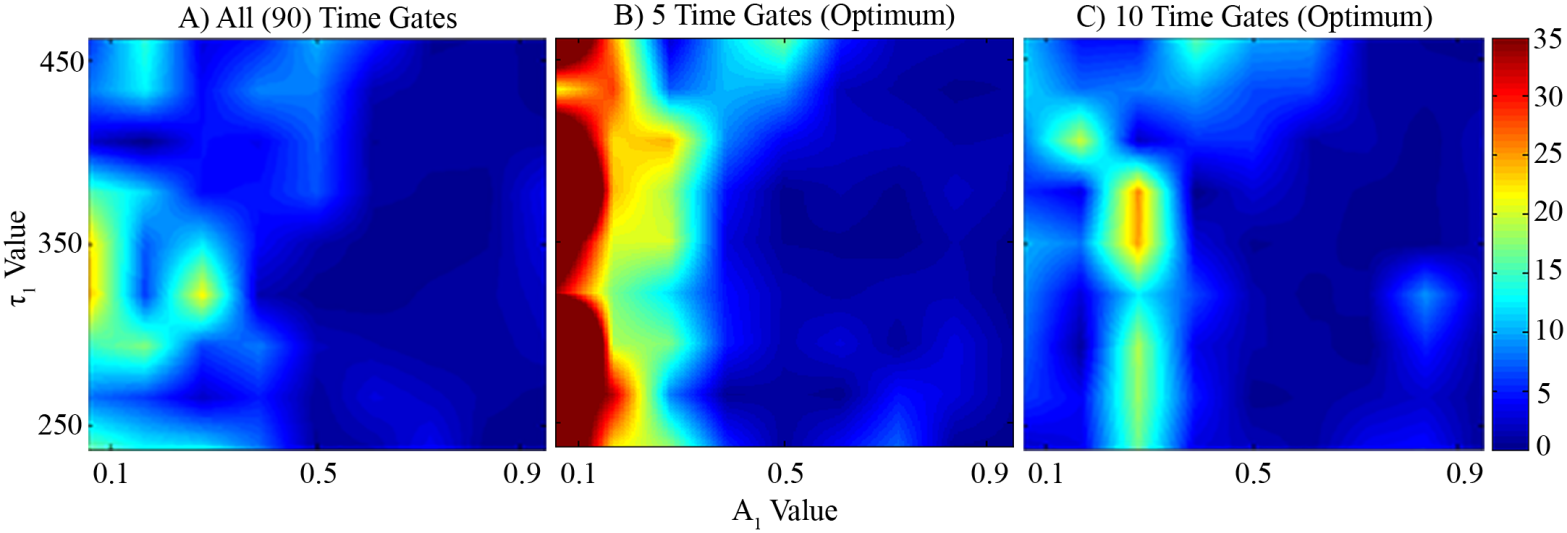
A comparison of parameter estimates using synthetic data across various experimental conditions. The average relative percent error in estimation of A_1_ across different parameter values using all (A), the 5 optimum (B) and the 10 optimum (C) time gates shown in Table 1 is shown. Average error across all parameter values increases from 5% (all) to 6% (10) to 12% (5) with the reduction in time gates. Data from this figure can be found in.csv format in Supporting Information S1 File (A), S2 File (B) and S3 File (C).

The noisy decay curves described in the Methods section were fit using MATLAB and either all of the time gates, five optimum time gates or the ten optimum time gates shown in Table 1. Each point in Fig 2 indicates the average relative error in estimation of A_1_ at that set of parameter values. The average error across the entire parameter space increases from 5% for all time gates to 6% when using only the optimum ten time gates to 12% when using only the optimum five time gates. Intuitively, the highest error occurs at smaller values of A_1_ where the quenched donor fraction has insignificant impact on the decay curves and smaller values of A_1_ result in higher relative error values. Interestingly, when using the optimum ten time gates the maximum error appears closer to A_1_ values of 0.3 rather than zero, though it is not immediately clear why this occurs. The simulations were repeated several times with varying numbers of iterations and the results were similar in all cases.

### *In Vivo* Results

**Fig 3:**
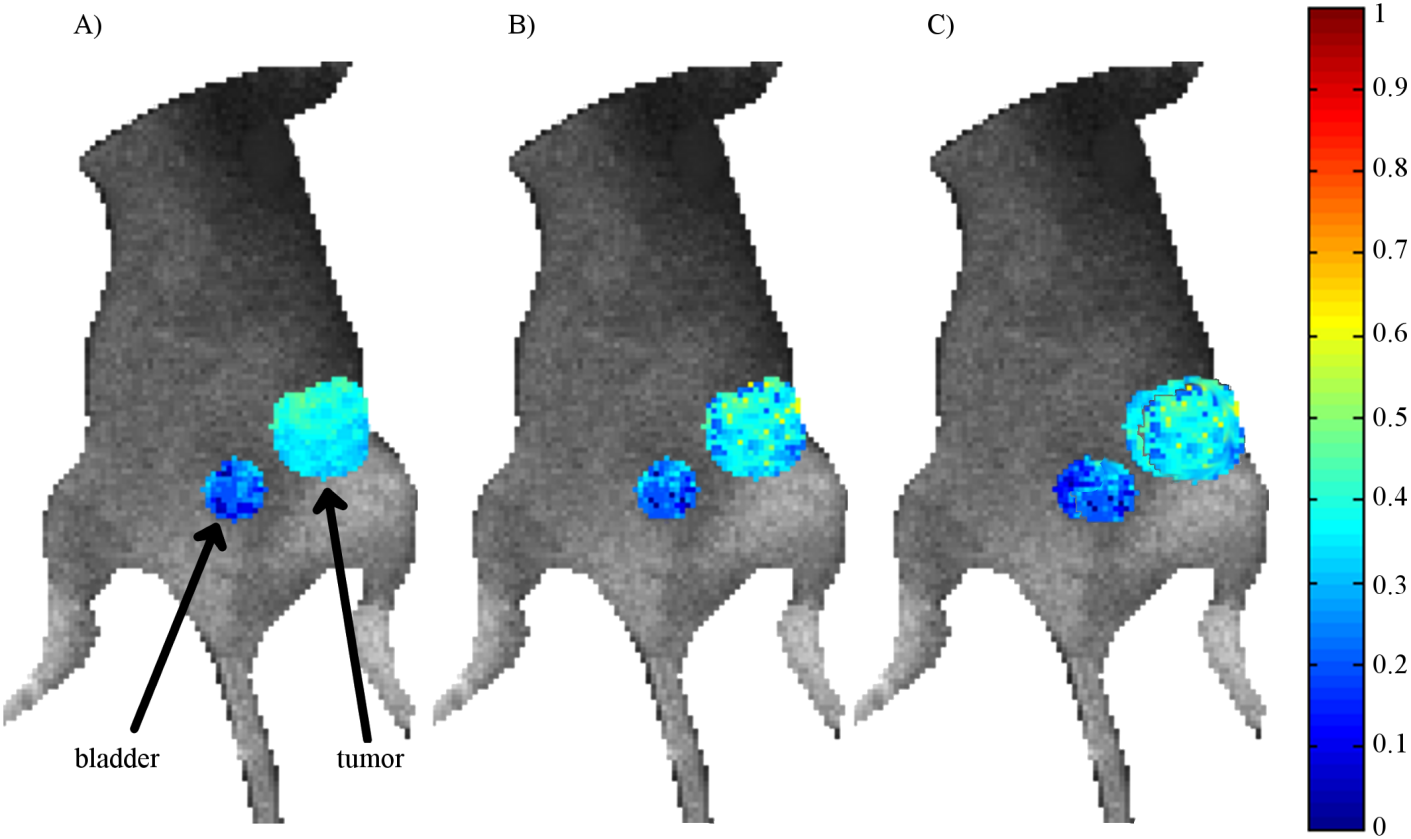
*In vivo* parameter estimates using all, 10 optimal or 10 evenly-spaced time gates. A comparison of *in vivo* estimates of quenched donor fraction (A_1_) in the bladder and tumor of a mouse. Estimates were calculated using either all (A), the optimal ten (B) time gates and 10 evenly-spaced (C) time gates overlaid on a bright field image of the mouse. Estimates of A_1_ are higher in the tumor in all cases and largely similar between the three sets of time gates. Data from this figure can be found in.csv format in Supporting Information S4 File (A), S5 File (B) and S6 File (C).

An *in vivo* experiment as described previously was also used to validate the optimization results. The quenched donor fraction (A_1_) in the tumor and bladder of the mouse were estimated and are reported in Table 3 as well as shown in Fig 3. A_1_ in this application represents the amount of transferrin on the endocytic pathway [60, 61]. This type of experiment is especially useful in drug development as the uptake of therapeutics can be quantified at different time points without the need to sacrifice the animal. Results using all the available time gates and only the optimal (or evenly-spaced) ten are very similar in each of the recorded locations and the donor fraction varies by less than 2% (6.8% relative error) in both the tumor and the bladder. Standard deviations are also similar at each location comparing the two sets of time gates. An additional comparison is made using ten evenly-spaced time gates (as performed in [19]) and results are similar. These results show that using only ten optimal time gates it is still possible to clearly distinguish between the bladder and the tumor tissue.

**Table 3:** *In vivo* comparison of parameter estimates. A comparison of *in vivo* estimates of quenched donor fraction using all the available time gates, the optimal 10 time gates reported in Table 1 and 10 evenly-spaced time gates. The estimates are similar for all cases and allow a clear distinction between the bladder and the tumor tissue.

**# of Time Gates**	**All**	**10 (optimum)**	**10 (even)**
**Bladder**	0.205 ± 0.07	0.219 ± 0.08	0.216 ± 0.09
**Tumor**	0.368 ± 0.04	0.363 ± 0.07	0.372 ± 0.05

### Discussion

The *in silico* results showed similar accuracy using the optimum ten time gates obtained herein, or using all the available time gates. Average estimation accuracy of A_1_ over all parameters values was decreased by only 1% from 5% to 6%. Using only five optimal time gates resulted in an average of 12% error, but estimates at small values of A_1_ were very poor. The chief benefit of employing the method presented herein is the reduced time and computational burden required to select time gates. This framework is able to converge to a solution for optimal time gates in seconds or minutes whereas the method mentioned previously using large, random trials [19] required hours or days.

The results for several different FRET pairs in Table 2, for example, were obtained in fewer than ten seconds in each case. In the worst case scenario, branch and bound methods will search the entire solution space to find the optimal values; however, in practice, the solutions are often obtained much more quickly. The solutions presented in Tables 1 and 2 were obtained in at most a few thousand iterations, which means only a minor fraction of the entire solution space was evaluated before obtaining the optimal solution. Further, because of the convex nature of the optimization problem, we can be assured of a global solution.

The *in vivo* experiment serves to validate the *in silico* results that similar accuracy is obtained when using all the available time gates or the optimal ten from Table 1. In this case evenly-spaced time gates performed comparably to the optimal set of time gates, though this is unlikely to be the case in each experiment; future work will investigate this question. The two organs selected in each case are clearly differentiated and have comparable average estimates and standard deviations. These data were acquired in simple wide-field illumination and detection transmission. In such configuration, full spatial and temporal data sets can be acquired in ≈40s (120-160 gates). Implementation of temporal data reduction as proposed herein could lead to acquisition times of ≈2-5s. Such fast acquisition times would enable fast, whole body imaging as well as high-throughput multi-well imaging [8, 64]. However, the most significant benefits of reduced data sets are expected to be achieved in tomographic applications [63]. Typically, *in vivo* tomographic acquisitions can take upwards of 30-45 minutes even in the case of simple cross-sectional imaging [65]. When combined with wide-field compressive implementations leveraging structured light illumination and detection [66, 67], the whole mouse example shown in Fig 3 can be acquired in less than 5 minutes. This is a significant reduction in time that would allow kinetics studies, the imaging of multiple biomarkers via spectral encoding and/or the imaging of multiple animals in relatively short acquisition times. The extension of our approach to tomographic data sets and model-based inverse problems [35, 68] will be conducted in the future.

Overall, the results from these experiments show that it is possible to reduce the total number of time gates acquired from 90 to 10 without significantly decreasing parameter estimation accuracy. This reduction in time gates in turn reduces the acquisition time of FLIM-FRET platforms by approximately an order of magnitude. This greatly strengthens the appeal of FLIM-FRET imaging applied to high-throughput and/or *in vivo* applications that are suffering from lengthy imaging times.

## Conclusions

As FLIM-FRET continues to be used in more complex imaging applications it is critical to develop experimental strategies that enable fast acquisition times. Previous methods used to determine optimal information content of FLIM-FRET data employed exhaustive search algorithms that took hours or days to complete. These methods were too computationally demanding to be applied to more complex applications such as multi/hyperspectral or tomographic acquisitions. In contrast to this, the experimental design method implemented in this paper has been applied effectively to the FLIM-FRET platform. Optimization results show the optimal time gates are clustered into three groups near the beginning, middle and end of the TPSF. These optimal points were validated using both *in silico* and *in vivo* experiments. These experiments suggested that it is possible to decrease the total number of time points acquired by nearly an order of magnitude with minimal loss in parameter estimation accuracy across various experimental conditions often encountered in NIR FLIM-FRET. This reduction in acquisition time allows more complex implementations such as high-content analysis, high-throughput screening or tomographic *in vivo* imaging to be completed within a few minutes. Additionally, the sensitivity analysis framework described herein is highly suited to complex problems and is easily augmented for future applications to other dimensions such as spectra.

## Supporting Information

S1 File Results for parameter estimation using 90 time gates. This is a.csv file containing the data used to create Fig 2A and calculate the average error reported.
(CSV)

S2 File Results for parameter estimation using 5 optimum time gates. This is a.csv file containing the data used to create Fig 2B and calculate the average error reported.
(CSV)

S3 File Results for parameter estimation using 10 optimum time gates. This is a.csv file containing the data used to create Fig 2C and calculate the average error reported.
(CSV)

S4 File *In vivo* results for parameter estimation using 90 time gates. This is a.csv file containing the data used to create Fig 3A and calculate the average error reported.
(CSV)

S5 File *In vivo* results for parameter estimation using 10 optimum time gates. This is a.csv file containing the data used to create Fig 3B and calculate the average error reported.
(CSV)

S6 File *In vivo* results for parameter estimation using 10 evenly-spaced time gates. This is a.csv file containing the data used to create Fig 3C and calculate the average error reported.
(CSV)
